# Managing Complexity: Exploring Decision Making on Medication by Young Adults with ADHD

**DOI:** 10.3390/pharmacy6020033

**Published:** 2018-04-19

**Authors:** Louise C. Druedahl, Sofia Kälvemark Sporrong

**Affiliations:** Social and Clinical Pharmacy Group, Department of Pharmacy, Faculty of Health and Medical Sciences, University of Copenhagen, 2100 Copenhagen, Denmark; sofia.sporrong@sund.ku.dk

**Keywords:** ADHD, decision making, medication, experience, interview, focus group, young adult, Denmark

## Abstract

Attention-deficit hyperactivity disorder (ADHD) causes difficulties with hyperactivity, impulsivity and inattention. Treatment of ADHD includes both medication and non-pharmacological options. Knowledge of treatment preferences by young adults with ADHD is sparse. The objective of this study was to explore the beliefs and experiences of young adults with ADHD related to their medication treatment decisions. Data were collected in Denmark in 2016 through a focus group and individual in-depth interviews. Conventional content analysis was used. Ten young adults with ADHD (22-to 29-year-old) participated. Three major themes were identified: (1) the patient’s right to choose concerning ADHD medicine; (2) the patient’s decision of whether or not to treat ADHD with medication; and (3) factors affecting the patient’s decision on whether to take ADHD medication or not. The latter theme contained 15 factors, which were distributed across three levels: individual, between-individuals, and societal. The dominant factors were increasing quality of life and improving oneself e.g., improving social skills. For counselling at the pharmacy and by prescribers, it is important to be aware of the different factors that affect young adult patients’ decisions on whether to take ADHD medication or not. This knowledge will aid to understand reasons for non-adherence and to determine appropriate treatment for the individual patient.

## 1. Introduction

Attention-deficit hyperactivity disorder (ADHD) is a disorder characterised by hyperactivity, impulsivity, and inattention [1]. The disorder is associated with impairment that can persist into adulthood [1]. A recent meta-analysis estimates the worldwide prevalence of ADHD to be 3.4% (CI 95%, 2.6–4.5) and estimates that 63 million people are affected [2]. In Denmark, ADHD is currently the most common psychiatric diagnosis among children and adolescents [3], and there is an increase in numbers of adults diagnosed [4]. In the period 2001–2011 the number of children and adolescents diagnosed with ADHD rose from about 1000 to about 8000, and correspondingly for adults from almost none to about 3000 [4].

Receiving a diagnosis and treatment might cause a profound impact on the individual. A diagnosis defines, predicts, and validates the experienced reality of the affected individual [5]. Psychiatric illnesses characterise deviances in behaviour relative to ‘normal’ behaviour [6], and the diagnosis of ADHD also relates to identity and self-image [7]. If the description of ADHD fits with one’s prior experiences in life, this can result in a new understanding of oneself in which a person internalises the diagnosis as part of his/her identity [7]. The cultural image of ADHD is also important for adolescents with regard to acceptance of the diagnosis [7]. Hence, an ADHD diagnosis offers clarity and the possibility of changes in self-perception, but it does not automatically resolve the difficulties experienced [8].

ADHD treatments include both medication and non-pharmacological options. Treatment with medication primarily consists of stimulants (e.g., methylphenidate or lisdexamfetamine) or non-stimulants (e.g., atomoxetine). In general, these compounds are considered effective for treatment of ADHD [9]. Non-pharmacological options include, amongst others, psychoeducation and cognitive behavioural therapy [10].

Non-adherence and discontinuation have been reported for children, adolescents and adults in relation to treatment of ADHD with medication [11,12,13]. For children and adolescents with ADHD, an important reason for parental discontinuation of ADHD-medication treatment was psychological adverse effects such as irritability or personality changes [13]. In addition, lack of perceived effect was associated with discontinuation [13]. For adults, discontinuation has been reported due to lack of or suboptimal effect, ‘not feeling like oneself’, and adverse effects both physical e.g., insomnia, and psychological such as ‘feeling besides oneself’ [12,14].

Following the transition from adolescence, young adults take on responsibility for important decisions in life, including medical decisions. Deciding on ADHD treatment with medication might be more difficult for young adults than for other age groups as important changes in attitude to treatment can occur from childhood to adulthood [7]. Treatment preferences by adults have been shown to be affected by a variety of aspects such as duration of effect during the day, and during afternoon/evening, absence of adverse effects, and dosage regimen [15,16]. However, there is a lack of knowledge about treatment preferences for young adults. The objective of this study was to explore the beliefs and experiences of young adults with ADHD related to their medication treatment decisions. This is in order to obtain a better understanding of the underlying rationales for taking or not taking medication and thus also adherence to treatment.

### The Setting

Medication is the first choice in the Danish national guidelines for the treatment of ADHD in adults, unless opposed by the patient [10]. Non-pharmacological treatments (psychoeducation and cognitive behavioural therapy) are to be initiated only if there is lack of effect by or intolerance of the medication [10]. However, non-pharmacological treatments in other sectors than the healthcare system can also be applied such as pedagogical or psychosocial care; these include counselling or assigning either a mentor or a special education teacher [17]. These options can be obtained for Danish citizens through the Danish welfare system if granted by the municipality of residence. In Denmark, most health-care services are free of charge. Prescription medication is gradually reimbursed depending on the annual costs for the individual patient. The annual cost is cumulated per purchase per citizen throughout the year and reimbursement per purchase can range from 0% up to 85% [18]. Only psychiatrists can prescribe ADHD medication.

## 2. Materials and Methods 

Two qualitative methods were used, semi-structured individual and focus group interviews.

### 2.1. Recruitment

The inclusion criteria were being a young adult aged 18–29 years, being a resident of Denmark being diagnosed with ADHD, and having been prescribed ADHD medication. Participants self-reported their ADHD diagnosis. Participants were recruited using a convenience sample approach by posting calls on the website of the Danish ADHD patient association as well as the association’s Facebook groups and pages. The association allowed posting information about the study on respective sites (without payment). Recruitment lasted February–August 2016. Upon contact, eligible participants were sent information about both the project and participation in the project. No reimbursement for participation was given.

### 2.2. Data Collection

An interview guide was developed, inspired by Brinkman et al. [19]; these authors interviewed adolescents in focus groups about decision making on ADHD medication in the U.S. In the present study, the interview guide contained three large topics: (1) purpose and thoughts about use of medication; (2) the perceived role of the young adults in decision making regarding ADHD medication; and (3) a Foucauldian perspective. The 3rd topic will be reported elsewhere [20]. Examples of interview questions can be seen in Table 1. Extensive probing was used in all interviews. All interviews were audio-recorded and transcribed verbatim. For the focus group, attention was devoted to familiarising the participants before initiating the discussion. Data collection was carried out from March to August 2016. All persons volunteering to be interviewed were included (convenience sampling). The focus group interview was held at the site of the University of Copenhagen, where the 1st author was moderator and the 2nd author was co-moderator. The individual interviews were held either in person, over the telephone, or using the Voice over Internet Protocol (VoIP), depending on what was possible and on the wishes of the respective interviewee. The 1st author conducted all individual interviews.

### 2.3. Data Analysis

A conventional content analysis was carried out [21]. Codes were inductively identified from the transcripts [21]. The transcripts were analysed separately by the authors to identify relevant data, i.e., beliefs or experiences related to or surrounding the topic of ADHD medication. Relevant data were thematised using paper and pen and thereafter compared between authors. The authors discussed the analysis at three meetings where they critically reflected on the themes and their content which resulted in clustering of the data into the structure presented in the results. The purpose of the meetings was to finalise the content analysis in consensus for rigor in the analysis and that the analysis reflected the data as a whole.

### 2.4. Ethics

No ethical approval was required for the study according to Danish law [22]; however, ethical considerations were met. Participants were informed about the study and assured of their anonymity as well as confidentiality. They all gave written informed consent to participate in the study, and their data were anonymised.

In the results section, all participants are given a pseudonym. All quotes were translated from Danish to English by the authors; quotes originating from the focus group interview are marked FGI.

## 3. Results

Ten persons participated in the study, including seven women and three men. Seven individual interviews and one focus group interview with three women and one man were conducted. One woman participated in both an individual interview and the focus group according to her own wishes. The duration of the focus group interview was 2 h and 7 min, and the length of the individual interviews ranged from 40 min to 1 h and 11 min. The age of the participants was 22–29 years (mean: 23.9 years), and their age at diagnosis was 13–29 years (mean: 20.1 years). All participants were diagnosed with ADHD; two specified the diagnosis as the inattentive subtype of ADHD. All respondents reported to be diagnosed by a psychiatrist. At the time of the interviews, seven interviewees were taking ADHD medication (various medications such as methylphenidate and lisdexamfetamine), and three were not (Jakob, Simone and Serhats), although one planned to restart a few months after the interview. All interviewees reported having used at least one type of ADHD medication. The employment situation of the interviewees varied, e.g., public welfare support, studying or working. Two interviewees said that they had previously had a substance-abuse problem with amphetamine, but both stopped before initiating ADHD medication treatment (methylphenidate and lisdexamphetamine). One of these interviewees spoke of the use of marijuana at social gatherings and in stressful situations, and a third interviewee reported regularly smoking marijuana to cope with symptoms of ADHD. These three interviewees did not differ from other interviewees in the identified themes.

The conventional content analysis of the combined data sets identified three major themes: (1) the patient’s right to choose ADHD medication as treatment; (2) the patient’s decision of whether or not to treat ADHD with medication; and (3) factors affecting the patient’s decision on whether to take ADHD medication or not.

### 3.1. The Patient’s Right to Choose ADHD Medication as Treatment

Nine out of ten participants expressed that it was their choice whether they wanted ADHD medication as a treatment for their ADHD or not. However, one interviewee said that her general practitioner (GP) had persuaded her to try psychotherapy before a second attempt at medication. Yet for a few participants, the first time they faced the decision regarding ADHD medication, they felt that the psychiatrist had made the choice for them, although they gained autonomy over the decision later.

“To begin with my choice about taking medication: I was 16 years old and I was told I had ADHD. The psychiatrist told me about it. And that [she] had sent a prescription to the pharmacy and that I should fill it/…/The only times where I will not take medication is during pregnancy” (Julie, FGI)

The interviewees expressed that they wished to openly discuss the decision-making about ADHD medication with their physicians. The respondents reported differences in the scope of the discussion about ADHD medication with the prescribing psychiatrist. For Alberte, she told about her psychiatrist as a sparring partner in her decision-making about treating ADHD with medication. The outcome of this sparring was described to be both periods with and without Alberte taking medication. Simone spoke about the need for her psychiatrist to explain both the effects and adverse effects of ADHD medication because the knowledge helped her decision making, but she also told about feeling an obligation towards her psychiatrist to take medication because the psychiatrist had spent time on her. Still, respondents stressed the importance of their right to choose whether to use ADHD medication or not.

“Well as a [health-care] professional, I think it is also important that you are able to see it from both sides, so you don’t influence the decision. But that you feel that or we feel that there is a choice” (Katrine, FGI)

### 3.2. The Patient’s Decision of Whether or Not to Treat ADHD with Medication

The decision to take ADHD medication as a treatment was regarded not as a permanent decision but as one that could be re-evaluated depending on, e.g., improvement in experienced symptoms, lack of effect of psychotherapy, or pregnancy. One participant argued that it was a reoccurring challenge to face this decision. The interviewees who did not take medication said that they initially started with medication because they hoped it would be the solution that could minimise the impact of their ADHD (e.g., improving ability to concentrate to allow taking the education one wants). However, the outcome of medication use was not the desired effect, which caused them to feel disappointment such as Serhats who told about how he hoped that medication would have improved what he termed his ‘social defects’. None of the interviewees used ADHD medication occasionally. Their decision-making involved weighing the advantageous and disadvantageous effects of ADHD medication use.

“If I had more adverse effects than positive effects, then I think I would probably consider how much I really needed it [the medication] and if I could live with the adverse effect or not” (Julie, FGI)

### 3.3. Factors Affecting the Patient’s Decision on Whether to Take ADHD Medication or Not 

The third theme contains 15 factors that were spoken of as advantages or disadvantages when considering taking ADHD medication, i.e., influencers of their decision on whether or not to take ADHD medication; see an overview of the factors in Table 2. The factors were spoken of both regarding the decision making to initiate treatment with ADHD medication as well as when evaluating if the participant wanted to continue to treat ADHD with medication. In the text, the factors that are mentioned are in italics. The 15 factors were distributed across three levels: individual, between-individuals (e.g., interacting with friends), and societal. Out of the 15 factors, 11 included both advantages and disadvantages, one factor included only advantages, and four factors included only disadvantages of taking medication. Below, the factors for the three levels will be described in more detail.

#### 3.3.1. Factors on the Individual Level

Nearly all interviewees spoke of *quality of life* in relation to their decision making regarding ADHD medication. For those taking medication, it was the means for them to achieve higher quality of life and enabling them to have a good life. However, those not taking medication also spoke of higher quality of life without medication despite the impact of ADHD.

“It is this [the medication] that makes me feel good” (Frederikke)

“Had I continued to take medication then, I think in the end I would just have sat and looked at a wall because it was the only thing I could do. Then there would not have been much to live for” (Simone)

The necessity of ADHD medication for ‘quality of life’ or alternatively as ‘vital for life’ was reflected upon during the focus group interview.

Alberte: “I think you also need to/…/be critical and it isn’t like it is a question of life and death, it isn’t”

Katrine: “It is for some, if you don’t get help”

Alberte: “That’s right, but that’s about quality of life. After all it’s not like you are a diabetes patient who…”

Katrine: “No, [it’s not like] ‘you die if you don’t take it’, I agree” (FGI)

The interviewees described *experience of oneself* as a way to evaluate the effects of ADHD medicine. The *experience of oneself* with medication varied a great deal from, e.g., ‘feeling better’/‘more like themselves and who they want to be’ to ‘becoming someone they did not want to be’.

”I don’t have that “bubbly” feeling or that I can’t be myself. I feel more like being in a bubble when I am not taking the medicine. (Katrine, FGI)

“I felt like I was flying around outside of my body when I took it [medicine]” (Simone)

The interviewees taking medication related their experienced change in behaviours and improvement of symptoms as both desirable and positive. Not feeling like they were on medication or were ‘drugged’ was emphasised as important for their feeling of self. The ADHD symptom of impulsivity was seen as something positive, but they felt unable to benefit from it when using medication. Several interviewees also expressed a feeling of obligation towards themselves to either use or avoid ADHD medication depending on their individual experiences when receiving ADHD medication as treatment, for example, ‘to get somewhere in life’ or ‘feeling okay’.

“Of course I have a choice, I can just decide not to [take medication]… But I also know, that then I won’t feel that well… So I basically don’t have a choice really. If I want to feel more or less okay, I probably have to [take medication]” (Heidi)

*Performance/Ambitions* was rarely spoken of by the participants not taking medication and was mentioned only in relation to the option of coping techniques to increase performance, with such techniques making it easier to handle ADHD symptoms. In contrast, *performance/ambitions* was noted as important to participants taking ADHD medication related to their experience of feeling better at performing (e.g., better at keeping their thoughts focused) or finding it possible to achieve their goals, e.g., to be able to study or generally ‘to get somewhere in life’. Frederikke described how she wanted to take an education, but to achieve that she needed to be able to remain seated and study. She told about her poor results from elementary school, and about how she now is improving those as an effect of taking medication. She pointed out that without medication this would not have been possible. She explained that with her improved results the next step would be to apply for admission to her dream education. In general, intake of medication was described as the respondents’ way to perform closer to the level of performance achieved by individuals without ADHD.

“One hopes that it (the medication) can add more structure to my daily life. That it perhaps can make it possible for me to manage some extra hours” (Rebekka)

“With the medicine in my body, I can come really far in my life, but without the medication, I’m afraid I’ll stall” (Julie, FGI)

“If I can’t get this education without getting help [medication], then I have to ask: Is it the right thing for me? Should I perhaps do something else with my life? Perhaps something where I am not under so much pressure and don’t need medication to get by” (Alberte, FGI)

“I don’t get super exams just because I take my Ritalin^®^. I just sort of normalise ‘I can be here, I can sit here and I can shut everything else out’” (Katrine)

The majority of the respondents spoke about *adverse effects*. These included palpitations, perspiration, reduced or loss of appetite, cold extremities, abdominal pains, insomnia, and changes in personality. A few respondents told of the impact on mood, where feelings of sadness or feeling depressed occurred 10–14 days after the initiation of ADHD medication. On this basis, Christian described how he had developed a pattern of discontinuation-initiation of treatment with ADHD medication drugs to avoid the undesired effect on his mood. Other respondents also mentioned autonomous dosage regulation to increase the benefits and avoid the adverse effects or both simultaneously. For some participants, the level of adverse effects did not counteract the beneficial effects. Almost all of the participants who said that they had either switched types of ADHD medications or stopped taking medication also explained that this was because of adverse effects.

“The first 14 days [with medication] were really, really nice, and one feels good. You could feel that something had happened. Then, when the adverse effects start, you kind of just live in your own hell” (Jakob)

“I spent a lot of time with a friend/…/and at the time I hadn’t seen him for about 14 days/…/, and when his mom opened the door, I only got to say hi to her when she asked, ‘did you get some new medicine?’ I wondered why she asked, and I replied, ‘no, but I stopped [taking medicine], why do you ask?’ She explained that it was because she hadn’t seen me smile since I started on the medicine” (Jakob)

The *safety/risk estimation* associated with taking ADHD medication was evaluated by several interviewees. *Safety/Risk estimation* was spoken of as potential future hazards in contrast to adverse effects that were only described as events that had occurred in the past. Views of the hazards of taking ADHD medicine were expressed as, e.g., medication harming the organs with long-term use, while others did not associate ADHD medication use with affecting the body or heart. Interviewees who currently took medication spoke about hazards. Several female interviewees identified pregnancy (when/if that would happen) as a reason for discontinuation, as they emphasised that medication in the body during pregnancy was dangerous. However, some participants felt that there was also danger associated with choosing not to take medication, as they argued that untreated ADHD could develop into other mental illnesses, such as schizophrenia or depression.

“The medication doesn’t harm your body; it’s not like you are more susceptible to heart [disease] or anything like that” (Julie, FGI)

“In the long run it [the medicine] can be harmful” (Frederikke)

*Reliance on effect* of ADHD medication was described by several interviewees, arguing that medication was a necessity for them. Julie described how she was unable to control herself without medication and she explained that she now relied on the effect of medication in order for her to live her life.

“I can’t relax unless I have my Ritalin^®^. That’s really what it is all about” (Katrine)

*Personal finances* were an issue for a few respondents due to the medication being too expensive. This caused one interviewee to discontinue medication treatment, but to start again when generics had been marketed and sold at a lower price. *Being natural* was spoken of in relation to not wanting medication as it was regarded as ‘chemicals’. Both participants taking ADHD medication and those not taking it used *coping* techniques. Those not currently taking medication strongly emphasised the importance of developing techniques to handle the impact from ADHD symptoms. The mentioned coping techniques were creating structure (calendar, weekly schedule, and making lunch for the next day in the evening) and becoming aware of one’s limits (how many tasks per day and mood).

“And then I was put on Strattera. But they cost 1500 [Danish kroner], which was too expensive” (Christian)

“It’s possible to reduce it [ADHD], which over time, I did. It’s possible, if you bother to spend a bit of time on getting to know yourself a bit” (Jakob)

#### 3.3.2. Factors on the between-Individuals Level

Interviewees told of *family/friends* being annoyed by them when not taking medication, which was argued to be a potential reason for difficulty in stopping taking ADHD medication. Their ADHD behaviours seemed to repulse other people and caused them to feel misunderstood by those in their surroundings. The interviewees expressed that people in their surroundings (GP/psychiatrist, family, friends) had an opinion or tried to affect their choice about ADHD medication.

“My [medicine] helps me to concentrate and also so I don’t piss everybody off” (Katrine, FGI)

“They [friends and family] actually liked me better without [medication]. Most of them said so. For sure, not one of them said that I should take it again” (Jakob)

A couple of interviewees pointed out that they had better *social skills* without medication, although others said that taking medication allowed them to be more present and have deeper conversations as well as relationships with their family and friends. Heidi explained that without medication she could not talk about her emotions, which resulted in occasional outbursts of anger, and how she felt that taking medication had improved this. Heidi described that not only herself benefitted from the effects of the medication, but also the people around her, from her family and friends to shop assistants in stores. Independent of whether interviewees were taking medication or not, they expressed that some individuals in their surroundings understood their choice but that some did not. The choice that gave the participants higher quality of life was also expressed to be the better solution for their surroundings and social life.

“People can better tolerate being in my company [when I take medication]” (Christian)

“Well, it’s bloody sad some of the things one does to people [while taking medication], you still feel bad about it today” (Jakob)

#### 3.3.3. Factors on the Societal Level

The *societal culture* in Denmark was identified as discouraging the use of ADHD medication. The interviewees claimed that the Danish culture was unaccepting of ADHD as a diagnosis, and that ADHD was portrayed as something fabricated. They saw Danish media as enforcers behind the scepticism about ADHD as a diagnosis and its treatment. Interviewees found these depictions of ADHD in society to be wrong, such as the view that ADHD medication is prescribed too easily. The wrong impression in the public was argued to be caused by a lack of understanding of how the mind works for a person with ADHD, which leads to a misunderstanding of ADHD behaviours. For example, Frederikke said that it was the medication that allowed her to take part in society, e.g., to vote in elections. People with ADHD were said to suffer from the media debate; interviewees expressed the outcome of this to be stigmatisation and, for some, not receiving proper drug treatment. Still, most interviewees said that they were not influenced by the public debate, though most told of being upset or angry by it, but that it made them more firm about their decision.

“And then it escalated in the media [negativity about the ADHD diagnosis]…. And it is bloody irritating because it affects my daily intake…. But I am like, nope! Damn it, I can’t let this influence me, because the symptoms are real.” (Katrine)

*Stigma/Taboo* was identified by participants both in relation to social life and in society. In society, this was related to feeling different from the typical depiction of having ADHD, such as documentaries showing unruly children. Regarding social life, some described ADHD medication as helping to avoid stigmatising social issues and situations. Stigma was related to both having the disorder and taking ADHD medication, but not necessarily concurrently. Stigma could be handled, e.g., by withholding information about having the disorder, taking medication until it was relevant to mention or telling only selected confidants. Several respondents experienced ADHD and ADHD medication as taboos; for some respondents, this also occurred within their families.

“I think it is a bit distasteful to say that I take medicine and psychiatric medication. It is still looked down on, I think” (Serhats, FGI)

“I believe that it is because you are afraid to get labelled and thereby stigmatised. Somehow that you are reluctant to [take medication]” (Alberte, FGI)

The *image of medication* for ADHD was by some participants perceived as a crutch and by others as evil.

“I don’t bloody feel like saying that I take three pills a day to anyone because it has become such a… such an evil thing, right” (Katrine)

Various *non-medication treatments* were described, e.g., psychoeducation or ADHD courses. A majority of the interviewees indicated that they wanted their treatment of ADHD to include a non-medication aspect while expressing the hope of being able to avoid medication. *Non-medication treatments* were preferable either alone or in combination with a treatment regimen. However, the participants argued that they lacked knowledge about non-medication approaches for the treatment of ADHD, and they desired that a psychiatrist should give this information to them. Rebekka described the consequence of this as leaving her to believe that medication was the only treatment that existed. Several interviewees identified *non-medication treatments* as unavailable. Three reasons were proposed to explain the difficulties in acquiring these treatments: (1) they are costly; (2) they can be difficult to be granted by the municipality; and (3) there can be disbelief about the presence of a disability caused by ADHD because it is an invisible illness, causing limited success in applications for social benefits.

“I would like to try it [computer-based training], but it is way too expensive” (Serhats, FGI)

## 4. Discussion

The main findings of this study are that these young adults with ADHD felt that they had a choice to make regarding treating ADHD with medication. This choice was their own, but many advantages and disadvantages needed to be considered in relation to this decision. Fifteen factors were found to influence the decision-making. These were distributed across three levels: the individual level, the between-individuals level, and the societal level.

**Figure 1:**
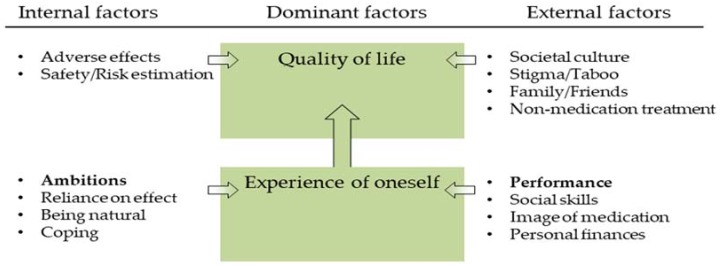
Model of factors relevant for decision making regarding medication by young adults with ADHD. Bold indicates factors coming across as dominant.

Counterbalancing the advantages and drawbacks of ADHD medication intake has been reported previously [12,19,23]. However, when interpreting the results of this study, a pattern was identified on how the 15 factors related to decision-making (Table 2) were hierarchically related to each other; see Figure 1. The hierarchal pattern was seen in the stories told by the individual interviewees/focus group participants. Dominant factors (*quality of Life* and *experience of Oneself*) seemed to be more ultimate goals also when other factors were discussed by the participants. This means that the factors weighed as either an advantage or disadvantage by influencing *experience of oneself* and *quality of life* depending on the context of the individual young adult. An example is a young adult taking ADHD medication and on one hand experiencing adverse effects which negatively affected his/her *quality of life.* However, on the other hand, if concomitantly experiencing improvement in *social skills* and *performance* these would allow the person to have a better experience of him-/herself and hence a better *quality of life*. The magnitudes of the different factors thus served as a foundation for the individual to decide if life is better with or without ADHD medication as treatment.

*Performance* and *ambitions* (marked in bold in Figure 1) seem to be more dominant than the other 12 factors. This is supported by previous results from interviews with young adults with ADHD [23,24,25,26,27]. *Experience of oneself* or ‘the sense of self’ was a dominant factor in the decision-making process, which is supported by several studies [12,23,24,25,27]. Davis-Berman and Pestello [24] also reported on factors impacting each other; specifically, they saw that *performance* and *ambitions* affect the *experience of oneself*. That is, an effect on *experience of oneself* could be tolerated when an individual had a need to perform which led to a consistent intake of ADHD medication. However, when there was no need to perform, e.g., during weekends or summer breaks from college, the individual took time off from using medication [24].

Compared to earlier studies [12,23,24,25,26,27,28,29,30,31,32], there are two factors that did not appear in the present study: shame [28], and difficulty with access to care (in England) [23]. The latter may not have been reported due to cultural differences in the health-care systems in England and Denmark.

The present decision-making model needs to be considered in light of these interviewees describing their current or previous ADHD medicine intake to be consistent and not situation-based, i.e., not used to, e.g., maximise concentration for exams, such as reported by Fleishmann and Kalinski [33]. The factors *performance* and *ambitions* are essentially different from the non-medical use of prescription stimulants for non-medical matters as has previously been reported to occur in this age group (see, e.g., Robitaille and Collin [34]).

A partial effect of ADHD medication was expressed by the participants and has also been reported for other adults with ADHD [32]. Choosing to take medication despite achieving only partial relief of symptoms could occur because (i) the drawbacks to taking medication are not substantial or (ii) the impact of ADHD symptoms is heavily affecting the individual so he/she develops a “high tolerance” for the barriers against taking medication. The partial relief of medication can also be a reason for the participants wanting to have non-medication treatments or a combination of treatments, as non-medication treatments might provide more relief from the experienced ADHD symptoms. However, an obstacle for participants in this study was lack of knowledge and access to this type of treatments.

The results allow for a deeper understanding of young adults’ beliefs of and experiences with taking ADHD medication, and how these impact considerations about adhering to medication treatment. Choosing to treat ADHD with medication seems to involve an active and reflexive decision-making process among young adults. This is important in a health-care setting, as it emphasises the significance of a dialogue about the choice of treatment of ADHD. Patients with ADHD want, can, and should have the opportunity to discuss their considerations on the topic. This is essential to be aware of during counselling both at the pharmacy and by the prescribing physician. The factors identified in this study affect patients’ decisions and could hence be used in order to provide better counselling both before the decision is made and when monitoring adherence and the effects of the medication. The choices made regarding ADHD medication, and the rationales underlying them, should not be left unaddressed in the interaction between health-care professionals and patients.

### Methodological Considerations

This study has some methodological aspects worth mentioning. First, other opinions about decision making regarding ADHD medication may exist as the sample was recruited as a convenience sample through a network of a patient organisation, therefore excluding those not in contact with such a network. Hence saturation may not have been reached. Also, the participants found the extra energy to take part in the project; some with a severe impairment caused by ADHD might not respond to recruitment attempts. Only three participants did not currently take ADHD medication compared to seven who did. This might have allowed advantages rather than drawbacks of taking ADHD medication to emerge. However, all interviewees described both advantages and disadvantages. In addition, the focus group included only one male and his voice could have been silenced by the female voices; however, all focus group participants were equally active.

## 5. Conclusions

This study shows that these Danish young adults with ADHD perceive the decision on ADHD medication to be their own and that they counterbalance advantages and drawbacks of taking ADHD medication when facing the decision. Fifteen factors, both internal and external, were identified as influencers of the decision regarding whether to take medication or not. The factors were related to each other in a hierarchical way, where *quality of life* and *experience of oneself* were dominant themes. In addition, *performance* and *ambitions* were more important than the other 12 factors. The knowledge of influencing factors is important for health-care professionals, such as prescribers and pharmacy staff, when counselling young adults with ADHD. This is in order to empower patients facing the complex decision on whether to take ADHD medication or not, and to help determine the most beneficent solution for the individual patient.

## Figures and Tables

**Table 1:** Examples of interview questions (translated from Danish). T = topic.

Examples of Interview Questions
“Why did you choose to take/not to take ADHD medication?” (T1) “What affects you, when you are facing the decision on whether to treat ADHD with medication?” (T1) “What considerations did you have when facing the decision?” (T1) “How do you perceive your role in relation to decision making regarding ADHD medication?” (T2) “How has this role changed over time?” (T2) “Who do you think benefits from your ADHD medication intake?” (T3)

**Table 2:** Overview of factors related to deciding whether to use attention-deficit hyperactivity disorder (ADHD) medication as a treatment.

Individual Level:	Between-Individuals Level:	Societal Level:
Quality of life Experience of oneself Performance/Ambitions Adverse effects Safety/Risk estimation Reliance on effect Personal finances Being natural Coping	Family/Friends Social skills	Societal culture Stigma/Taboo Image of medication Non-medication treatment
